# DHA Oral Supplementation Modulates Serum Epoxydocosapentaenoic Acid (EDP) Levels in Breast Cancer Patients

**DOI:** 10.1155/2019/1280987

**Published:** 2019-03-05

**Authors:** Alessio Molfino, Maria Ida Amabile, Luana Lionetto, Alessandra Spagnoli, Cesarina Ramaccini, Alessandro De Luca, Maurizio Simmaco, Massimo Monti, Maurizio Muscaritoli

**Affiliations:** ^1^Department of Translational and Precision Medicine, Sapienza University of Rome, v.le dell'Università 37, 00185 Rome, Italy; ^2^Department of Surgical Sciences, Sapienza University of Rome, v.le Regina Elena 324, 00161 Rome, Italy; ^3^Department NESMOS, Sapienza University, Analytical Laboratory Unit, Sant'Andrea Hospital, via di Grottarossa, 00135 Rome, Italy; ^4^Department of Public Health and Infectious Diseases, Sapienza University of Rome, p.le Aldo Moro 5, 00185 Rome, Italy

## Abstract

**Introduction:**

The omega-3 polyunsaturated fatty acids, as docosahexaenoic acid (DHA), are considered mediators regulating the resolution of inflammation during cancer and may be associated with better outcomes. Epoxydocosapentaenoic acids (EDPs), metabolites of the DHA, are hypothesized to be responsible for some beneficial effects. In the present study, we aimed to assess the circulating 19,20-EDP levels in breast cancer (BC) patients and in healthy controls before and after DHA oral supplementation and the potential differences in the DHA conversion in 19,20-EDPs between patients with different BC presentations.

**Methods:**

BC patients and healthy controls were supplemented with DHA (algal oil) for 10 days (2 g/day). Blood samples were collected at baseline (T0) and after supplementation (T1) to assess EDP (19,20-EDP) serum levels by liquid chromatography spectrometry.

**Results:**

33 BC patients and 10 controls were studied. EDP values at T0 were not different between patients and controls. At T1, we found an increase in 19,20-EDP levels in BC patients (*P* < 0.00001) and in controls (*P* < 0.001), whereas no differences in 19,20-EDPs were present between the two groups; when considering the type of BC presentation, patients with BRCA1/2 mutation showed lower 19,20-EDPs levels with respect to BC patients without the mutation (*P* = 0.03). According to immunohistochemical subtype, luminal A-like BC patients showed at T1 higher 19,20-EDP levels compared to nonluminal A (*P* = 0.02).

**Conclusions:**

DHA oral supplementation was associated with increased 19,20-EDP serum levels in BC patients, independent of the type of BC presentation, and in controls. Patients carrier of BRCA1/2 mutation seem to possess lower ability of DHA epoxidation, whereas luminal A-like BC patients showed higher EDP conversion. This behavior should be tested in a larger population.

## 1. Introduction

Polyunsaturated fatty acids (PUFAs) of the omega-3 series are essential nutrients whose dietary intake, with food and/or supplements, is associated with well-documented health benefits [1, 2]. Omega-3 PUFAs, primarily found in dietary fish oils and in plants, are substrates able to modulate and limit inflammatory responses, likely those that underlie mechanisms of chronic diseases such as cancer [3], including breast cancer (BC) [4].

The omega-3 PUFAs, docosahexaenoic acid (DHA) and eicosapentaenoic acid, may be considered modulators of the mechanisms regulating the onset, prolongation, and resolution of inflammation and, therefore, are believed to be protective against inflammatory response [1].

Metabolites of DHA, named as epoxydocosapentaenoic acids (EDPs), deriving from the conversion of DHA by cytochrome P450 epoxygenases, are suggested to be responsible for some of the beneficial effects attributed to omega-3 PUFAs and omega-3-rich foods, such as fish oil, and to mediate some of the health-promoting effects of DHA [5].

Evidences showed that omega-3 fatty acid metabolites and mainly EDPs mediate several beneficial effects in chronic conditions, including chronic pain and angiotensin II-dependent hypertension, by anti-inflammatory properties, and in kidney disease [6–10].

Experimental studies have documented that specific EDPs - 16,17-EDP and, in particular, 19,20-EDPs - are potent mediators in suppressing inflammation and inhibiting angiogenesis, endothelial cell migration, endothelial cell proliferation, and the growth and metastasis of human breast and prostate cancer [6, 11, 12].

Consumption of omega-3 fatty acid-rich diets raises the circulating and tissue levels of 19,20-EDPs in animals and in humans, which is the most prominent change in the profile of PUFA metabolites determined by dietary omega-3 fatty acids [13–15]. Therefore, it is reasonable to think that the metabolism of DHA to 19,20-EDPs may be responsible, at least in part, for some of the beneficial effects referred to dietary omega-3 fatty acids.

As previously shown [5, 16], 19,20-EDP is the most abundant EDP regioisomer detected *in vivo* with potential positive effects in cancer. In addition, data suggest that in BC patients carrying BRCA1 and BRCA2 mutations, a lipid and metabolite dysregulation is present [17].

In this perspective, we aimed to assess the circulating 19,20-EDPs levels in BC patients and in healthy controls and the potential differences in the DHA conversion in 19,20-EDPs between sporadic BC patients and BC patients with a family history of breast malignancy, either positive or not for BRCA1/BRCA2 gene mutation.

## 2. Subjects and Methods

This was an interventional, spontaneous, single-center, controlled study performed on patients from the Department of Surgical Sciences, Sapienza—University of Rome, Italy. After approval of the local Ethics Committee and after obtaining written informed consent from each participant, women with diagnosis of BC and healthy women without personal and/or family history of BC, participating to a study conducted to observe changes in DHA levels and omega-3 index before and after DHA oral supplementation [18], were considered. All procedures were in accordance with the ethical standards of the Helsinki Declaration issued in 1975 and later amendments. Exclusion criteria were self-reported consumption of omega-3 PUFA supplements, omega-3 PUFA-supplemented foods in the previous 6 months, and other neoplastic diseases, as previously described [18]. We previously demonstrated that a sample size of 33 BC patients and 10 controls was able to show significant changes in DHA circulating levels and omega-3 index [18]. Therefore, the same study size (and the same population) was used for the aims of the present investigation.

### 2.1. Breast Cancer Patients and Healthy Controls

We enrolled breast cancer patients at their first diagnosis, before starting any anticancer treatment. We recorded participants' demographic and anthropometric characteristics (age, weight, height, body mass index, and body weight change over the prior 6 months), the presence/absence of comorbidities (i.e., hypercholesterolemia, hypertriglyceridemia, and diabetes), and serum metabolic and nutritional biomarkers. Histological diagnosis, tumor staging, and a detailed medical history were collected. According to international classification, which is based on the familiar and past medical history, the participants were divided into sporadic (S) group, including BC patients without family history of breast malignancy; familiar (F) group, including BC patients with BC familial history, but negative for BRCA1 or BRCA2 gene mutation; and mutated (M) group, including BC patients with documented BRCA1 or BRCA2 gene mutation [18]. We also considered healthy age- and body mass index-matched women, serving as the control group (C) [18]. The questionnaire on participants' self-reported dietary habits was administered focusing on the consumption of seafood and its frequency [19], allowing to identify “low or good seafood consumer” [18].

### 2.2. DHA Oral Supplementation

We considered women enrolled in a study consisting in assuming 2 g of DHA per day in the form of strawberry-flavored algal oil syrup (from Schizochytrium sp. microalgae) containing omega-3 fatty acids >46% of the total fatty acids (DHA in the triglyceride form) (Richoil® syrup, provided free of charge by DMF Dietetic Metabolic Food, Limbiate, Italy) for 10 consecutive days [18]. Moreover, during those days the participants were prescribed to maintain a standard normo-balanced diet and the usual physical activity level. The participants had a reference telephone number to contact for discussing any difficulties during the supplementation period and to ensure compliance.

### 2.3. Blood Sample Collection and Epoxydocosapentaenoic Acid Assay

Blood samples were obtained on fasting from all the participants at baseline (T0) and after the 10 days of oral DHA supplementation (T1). Aliquots of serum were stored at -80°C until analysis.

Epoxydocosapentaenoic acid serum levels were assessed by a liquid chromatography–tandem mass spectrometry method. Twenty microliters of serum samples were added to sixty microliters of cold methanol 100%. After extensive vortex (60 sec), samples were maintained at 4°C for ten minutes and then centrifuged at 13,000 revolutions per minutes (rpm) for 15 minutes. Fifty *μ*L of supernatant was directly transferred to an autosampler vial for the injection into the chromatographic system. Ultra-performance liquid chromatography (UPLC) analysis was performed with a Sciex Liquid Chromatography System series ExionLC (AB Sciex, Ontario, Canada). Chromatographic separation was performed using a reverse-phase column (100 × 2.1 mm, Kinetex Biphenyl, 2.6 *μ*m, 100 Å pore size; Phenomenex, Torrance, USA). The column was maintained at 60°C. The mobile phases were UPLC-grade water (eluent A) and methanol (eluent B); the elution was performed at a flow rate of 600 *μ*L/min, using an elution gradient as follows: firstly 0.5 min with 5% eluent B and 1 min of linear gradient to 95% eluent B, followed by an additional period of 0.5 min in isocratic conditions and finally 1 min of 5% eluent B. The injection volume was 5 *μ*L, and the total run time analysis was 3 minutes. Mass spectrometry was performed on a 5500 triple quadrupole system (QTRAP Sciex, Ontario, Canada) equipped with an atmospheric pressure chemical ionization source (AB Sciex). The detector was set in negative mode. The Q1 and Q3 quadrupoles were tuned for the unit mass resolution. The instrument was set in the multiple-reaction monitoring mode. Mass spectrometer parameters were optimized to maximize sensitivity for each transition (Table 1). Data were acquired and processed with Analyst 1.7.0.

### 2.4. Statistical Analyses

Patients' characteristics were described using *mean* ± *standard* deviation (SD) for continuous normally distributed variables, including EDP levels, and separately by participant group, and percent for dichotomous variables. EDP values were nonnormally distributed and therefore transformed to the natural log (Ln) and one-way analysis of variance (ANOVA) and Student's *t*-test were used to evaluate differences among groups. R version 3.5.1 was used as statistical software.

## 3. Results

### 3.1. Participants' Characteristics

Baseline characteristics of the participants are reported in Table 2. In summary, a total of 33 BC patients and 10 healthy women were studied at baseline, well tolerated the daily doses of oral DHA, and completed the supplementation for 10 days, as previously described [18]. The participants were distributed as follows: 10 BC patients in the S group, 12 BC patients in the F group, 11 BC patients in the M group, and 10 women in the C group. Mean age was 47.3 ± 8.9 years for BC patients and 48.3 ± 5.66 years for the C group.

### 3.2. Epoxydocosapentaenoic Acid Levels at Baseline

We initially tested the potential presence of EDPs in the supplemented algal oil syrup, and none of them were detected.

At T0, no significant differences were observed in 19,20-EDP levels between BC patients and controls (*P* = 0.43), neither between each group of BC patients (S, F, and M groups). No association was found between 19,20-EDP levels and seafood dietary habits.

### 3.3. Epoxydocosapentaenoic Acid Levels after DHA Supplementation

**Figure 1:**
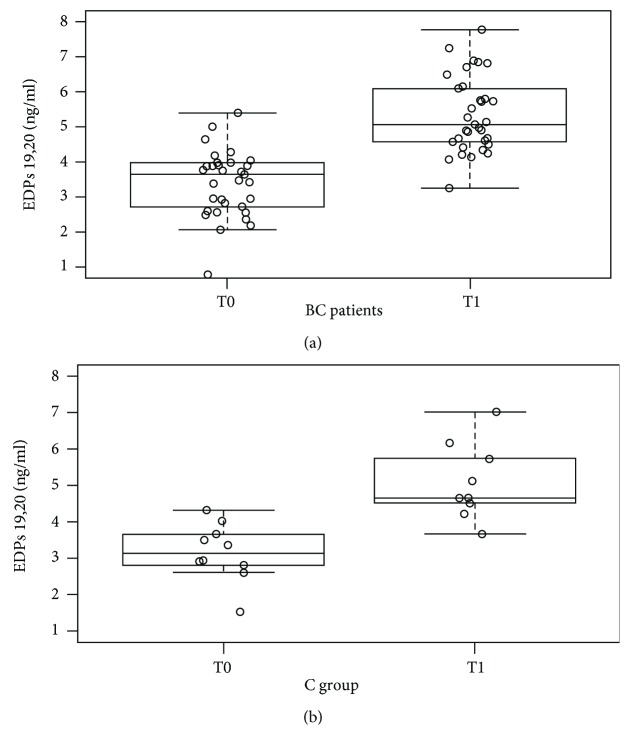
(a) Whisker plot of 19,20-EDP levels (ng/mL) in BC patients at baseline (T0) and after supplementation (T1). 19,20-EDP levels were higher at T1 with respect to T0 (*P* < 0.00001). Abbreviations: EDPs: epoxydocosapentaenoic acids; BC: breast cancer. (b) Whisker plot of 19,20-EDP levels (ng/mL) in C group at baseline (T0) and after supplementation (T1). 19,20-EDP levels were higher at T1 with respect to T0 (*P* < 0.001). Abbreviations: EDPs: epoxydocosapentaenoic acids; C group: control group.

After DHA supplementation (T1), 19,20-EDP levels significantly increased in BC patients (*P* < 0.00001) (Figure 1(a)) and in controls (*P* < 0.001) (Figure 1(b)). This significant change was confirmed in all the BC subgroups (*P* ≤ 0.001). Moreover, we did not find differences in 19,20-EDP levels between BC patients (S, F, and M groups) and controls (Table 2).

**Figure 2:**
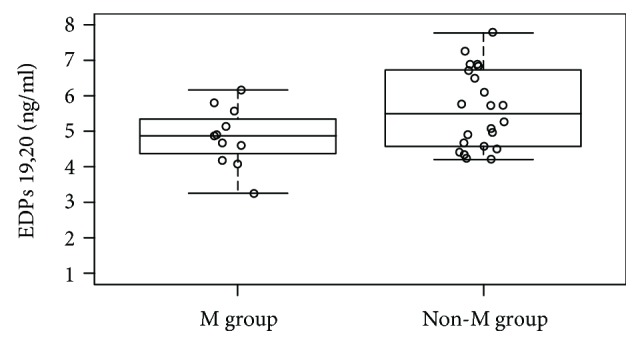
Whisker plot of 19,20-EDP levels (ng/mL) after supplementation (T1) in BC patients BRCA 1/2 mutation carrier (M group) and BC patients without the mutation (non-M group). 19,20-EDP levels were lower in the M group with respect to the non-M group (4.84 ± 0.82 vs 5.61 ± 1.10, *P* = 0.03). Abbreviations: EDPs: epoxydocosapentaenoic acids; BC: breast cancer.

Considering that we previously found that the M group presented a different DHA incorporation after oral supplementation compared to the other BC patients [18], we specifically analyzed 19,20-EDP levels in BRCA 1/2 mutation carriers vs the other BC patients without the mutation (*n* = 22), observing that 19,20-EDP levels (ng/dl) were lower in the M group (4.84 ± 0.82 vs 5.61 ± 1.10*P* = 0.03) (Figure 2).

As for baseline, no association was seen between 19,20-EDP levels and seafood dietary habits.

### 3.4. Epoxydocosapentaenoic Acid Levels according to Immunohistochemical BC Subtype

Stratifying BC patients according to immunohistochemical subtype, the majority of BC patients (*n* = 17, 52%) presented as luminal A, 6 as luminal B, 6 as triple negative, and 4 patients as Her2-positive. At T0, no significant differences were observed in 19,20-EDP levels between the different immunohistotypes.

**Figure 3:**
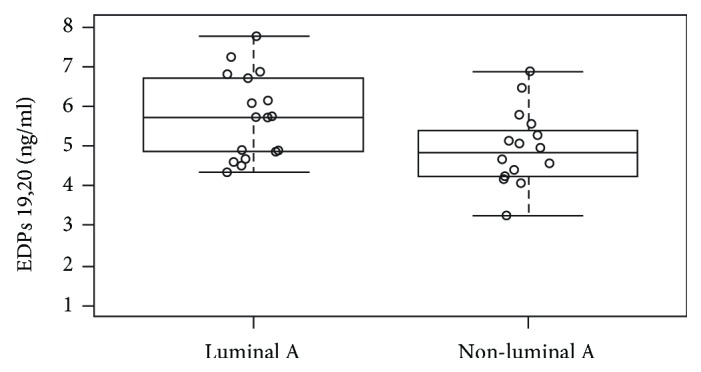
Whisker plot of 19,20-EDP levels (ng/ml) after supplementation (T1) in BC patients stratified by luminal A BC subtype or non-luminal A BC subtype. 19,20-EDP levels were higher in luminal A subtype with respect to non-luminal A subtype (5.75 ± 1.06 vs 4.93 ± 0.93, *P* = 0.02). Abbreviations: EDPs: epoxydocosapentaenoic acids; BC: breast cancer; T1: after 10 days of DHA oral supplementation.

At T1, we found that 19,20-EDP levels (ng/mL) in luminal A patients were significantly higher with respect to patients with other immunohistotypes (5.75 ± 1.06 vs 4.93 ± 0.93; *P* = 0.02) (Figure 3).

## 4. Discussion

Our data show that DHA oral supplementation in BC patients was able to change not only the circulating DHA levels and omega-3 index [18] but also 19,20-EDP serum levels, which have been correlated with cancer growth and progression [14]. In particular, by a 10-consecutive day DHA algal oil (2 g/day) oral supplementation, we found a significant increase in 19,20-EDP serum levels in BC patients and in healthy women (control group). Moreover, in each of the 3 BC groups (S group, F group, and M group), we observed a significant increase in EDP serum levels. However, no significant changes from baseline in 19,20-EDP levels were documented between BC groups and controls.

Our results, obtained using an easy-to-perform and well-tolerated supplementation, show the importance of modifying a specific metabolic profile (lipid metabolites) that might have an impact on the prognosis, and possibly on the therapeutic response, in BC patients. This may have potential implications in specific BC presentations, such as patients carrying BRCA 1/2 mutation, where the therapeutic options may be limited or with possible lower efficacy. In fact, our data showed that, after DHA oral supplementation, 19,20-EDP levels were lower among the M group with respect to the other BC patients. However, due to the small number of patients in the M group, these results should be further confirmed given the high heterogeneity of patients with BC, whose metabolic characteristics, which might be related to the disease progression and likely to a different therapeutic response, may profoundly differ depending on the type of BC presentation and/or immunohistotype. In this respect, we also found that patients presenting with luminal A subtype showed, after supplementation, higher 19,20-EDP levels compared to patients presenting with a nonluminal A subtype.

This result appears interesting considering that luminal A is the most favorable BC subtype [20], and no data are available in the literature on the possible relation between epoxides and prognosis in this clinical setting. However, serum 19,20-EDP levels of both luminal A and non-luminal A BC subtype did not differ from controls, although the sample size for this analysis is very small limiting its interpretation.

Metabolic derangements are common during BC [1]. Data obtained among patients presenting as BRCA mutation carriers indicate that the prevalence of metabolic disorders, in particular insulin resistance, is high [21]. Low insulin sensitivity and high diabetes rate have been observed in a 15-year follow-up in a group of BRCA mutation carriers who developed BC [21]. Considering that obesity and greater BMI values are modifiable risk factors for diabetes in BC patients with BRCA mutation, attention to novel metabolic and nutritional interventions, including omega-3 PUFA supplementation, should be implemented [4].

Previous clinical studies showed an increased formation of EDPs in humans upon DHA supplementation, indicating that our results are directly correlated with the effects of DHA oral intake [16, 22, 23]. In particular, Fischer et al. showed that EPA/DHA supplementation for 8 weeks doubled the levels of all regioisomeric DHA-derived metabolites, including 19,20-EDPs [24]. This behavior was confirmed by the results of our study.

Supplementation of DHA increases the levels of EDPs in most organs [5], and compared with other PUFA metabolites, EDPs are at least one thousand times more potent in terms of anti-inflammatory modulation [25]. These results further argue that EDP levels upon DHA supplementation cause potential multiple beneficial effects [21, 25].

Chronic inflammation is known as a potent contributor of BC growth and progression, playing a major role in the neoplastic process. Although hormonal modifications seem to play a driving role in breast carcinogenesis, the increased cytokine production may be a clinically relevant feature in BC [4]. However, how this condition has an impact on treatments' efficacy has to be better elucidated.

Several studies evaluated the effects of the supplementation of omega-3 PUFAs, including DHA, in resolving the inflammatory process [1, 2]. More importantly, this therapeutic strategy had a positive effect on patients' prognosis [4, 26]. Zhang et al. demonstrated that 19,20-EDP inhibits angiogenesis *in vitro* and *in vivo* and primary tumor growth [11]. To determine whether the anticancer effect was obtained from 19,20-EDP or from its metabolite derived by soluble epoxide hydrolase, the 19,20-dihydroxydocosapentaenoic acid (19,20-DiHDPA), the authors tested the effect of 19,20-DiHDPA on Met-1 tumor growth. The continuous infusion of 19,20-DiHDPA in mice did not have any effect on tumor growth, confirming that the anticancer effect was not from this diol metabolite [11]. Additional evidences indicate that inhibition of soluble epoxide hydrolase prevents vascular damages mediated by 19,20-EDPs [11]. However, in our clinical setting, information on how much DHA supplementation is necessary to reach an effective EDP concentration is not available.

Although our study involved a homogeneous population of BC patients from a single breast cancer unit, it presents several limitations. Our cohort of participants is small, and the BC patients involved may not be representative of larger BC patients' population, possibly presenting a high individual intervariability. The limited sample size of each subgroup of participants might have reduced the possibility of identifying association between patients' characteristics and EDP levels.

Moreover, we believe that a relevant aspect that should be further evaluated is the association between EDP levels and prognosis in BC patients.

## 5. Conclusions

The prevention and treatment of BC represent a relevant public health issue, and research on the relationship between BC, diet, metabolic intervention, and lifestyle should be implemented.

The human data that we obtained should be confirmed in a larger population and, more importantly, evaluated in a longitudinal fashion related to patients' outcomes.

## Figures and Tables

**Table 1:** Mass spectrometry parameters.

Precursor Ion *(m/z)*	Fragments *(m/z)*	DP	EP	CE	CXP
343.3	299.1	-95	-5.5	-13.3	-9.2
	285.1	-95	-5.5	-14.8	-10.7
	241.3	-95	-5.5	-15.1	-11.0

*m/z*: mass/charge ratio; DP: declustering potential; EP: entrance potential; CE, collision energy; CXP, collision cell exit potential.

**Table 2:** Participants' characteristics.

All participants *N* = 43	S group *N* = 10	F group *N* = 12	M group *N* = 11	C group *N* = 10
Age, years	49.4 ± 9.8	47.92 ± 8.46	44.82 ± 9.59	48.3 ± 5.66
Body weight, kg	62.8 ± 12.43	69.42 ± 11.87	58.91 ± 9.44	60.6 ± 7.4
BMI, weight (kg)/height^2^ (m)	23.68 ± 4.08	25.61 ± 4.52	22.32 ± 2.65	23.68 ± 2.93
Cholesterol, mg/dL	203.2 ± 31.36	210.09 ± 22.33	206.75 ± 35.57	217.7 ± 31.77
Glycemia, mg/dL	96.2 ± 8.85	92 ± 8.10	94.33 ± 12.26	88.72 ± 9.93
Comorbidities^#^ (y/no)	3/7	3/9	2/9	1/9
Ln EDPs (ng/mL) at T0	3.44 ± 0.74	3.64 ± 1.22	3.16 ± 0.72	3.16 ± 0.79
Ln EDPs (ng/mL) at T1	5.84 ± 1.05	5.41 ± 1.15	4.84 ± 0.82	5.03 ± 0.99

^∗^Data are shown as mean ± SD. *P* values are not significant for the patients' characteristics shown between groups. Abbreviations include: S: sporadic group; F: familiar group; M: mutated group; C: control group; BMI: body mass index; EDPs: epoxydocosapentaenoic acids. ^#^Dyslipidemia and type 2 diabetes.

## Data Availability

The data, including laboratory analyses, used to support the findings of this study are restricted by our Local Ethics committee [Azienda Policlinico Umberto I, Rome, Italy] in order to protect patient privacy. Data are available from Prof. Maurizio Muscaritoli (senior author, email: maurizio.muscaritoli@uniroma1.it) for researchers who meet the criteria for access to confidential data.
